# CCR2 improves homing and engraftment of adipose-derived stem cells in dystrophic mice

**DOI:** 10.1186/s13287-020-02065-z

**Published:** 2021-01-07

**Authors:** Liang Wang, Huan Li, Jinfu Lin, Ruojie He, Menglong Chen, Yu Zhang, Ziyu Liao, Cheng Zhang

**Affiliations:** 1 Department of Neurology, The First Affiliated Hospital, Sun Yat-sen University, No. 58 Zhongshan Road 2, Guangzhou, 510080 GD China; 2 National Key Clinical Department and Key Discipline of Neurology, Guangdong Provincial Key Laboratory of Diagnosis and Treatment of Major Neurological Diseases, No. 58 Zhongshan Road 2, Guangzhou, GD 510080 China; 3 Department of Neurology, Guangzhou Overseas Chinese Hospital, No. 613 Huangpu Road, Guangzhou, GD 510630 China

**Keywords:** Dystrophinopathy, Dystrophic mouse, Adipose-derived stem cell, Homing, CCR2

## Abstract

**Background:**

Dystrophinopathy, a common neuromuscular disorder caused by the absence of dystrophin, currently lacks effective treatments. Systemic transplantation of adipose-derived stem cells (ADSCs) is a promising treatment approach, but its low efficacy remains a challenge. Chemokine system-mediated stem cell homing plays a critical role in systemic transplantation. Here, we investigated whether overexpression of a specific chemokine receptor could improve muscle homing and therapeutic effects of ADSC systemic transplantation in dystrophic mice.

**Methods:**

We analysed multiple microarray datasets from the Gene Expression Omnibus to identify a candidate chemokine receptor and then evaluated the protein expression of target ligands in different tissues and organs of dystrophic mice. The candidate chemokine receptor was overexpressed using the lentiviral system in mouse ADSCs, which were used for systemic transplantation into the dystrophic mice, followed by evaluation of motor function, stem cell muscle homing, dystrophin expression, and muscle pathology.

**Results:**

Chemokine-profile analysis identified C–C chemokine receptor (CCR)2 as the potential target for improving ADSC homing. We found that the levels of its ligands C–C chemokine ligand (CCL)2 and CCL7 were higher in muscles than in other tissues and organs of dystrophic mice. Additionally, CCR2 overexpression improved ADSC migration ability and maintained their multilineage-differentiation potentials. Compared with control ADSCs, transplantation of those overexpressing CCR2 displayed better muscle homing and further improved motor function, dystrophin expression, and muscle pathology in dystrophic mice.

**Conclusions:**

These results demonstrated that CCR2 improved ADSC muscle homing and therapeutic effects following systemic transplantation in dystrophic mice.

## Background

Dystrophinopathy is a common X-linked recessive neuromuscular disorder characterised by progressive muscle wasting and weakness [1]. Its pathogenic gene is *DMD*; mutation in *DMD* causes absence of dystrophin, a protein located on the cytoplasmic surface of the sarcolemma [2]. Loss of dystrophin function results in sarcolemma instability, which causes pathological alterations in muscles, such as necrosis, repeated cycles of myofibre regeneration, and fibrosis [2]. According to different phenotype severities, dystrophinopathy can be divided mainly into Duchenne muscular dystrophy (DMD) and Becker muscular dystrophy (BMD). There remains no effective therapy to cure this disease, and patients usually lose ambulation and die due to respiratory or cardiac complications in the advanced stage [1, 3]. Therefore, development of an effective treatment strategy for dystrophinopathy is critically important.

Stem cell therapy represents a promising treatment for dystrophinopathy. Adipose-derived stem cells (ADSCs) are a type of mesenchymal stem cell (MSC) isolated from adipose tissue and are among the most commonly used donor cell types due to their relatively simple collection and low immunogenicity [4]. ADSC engraftment is an effective treatment modality in dystrophic mice [5–8]. There are two main methods of ADSC transplantation: systemic and local. Although the latter shows better therapeutic efficacy [6, 8], systemic transplantation is more suitable for dystrophinopathy, because most of the muscles in the body are injured [3], and it is impossible to inject stem cells into every injured site. In dystrophinopathy, there are two main routes for systemic transplantation: intraarterial delivery and intravenous delivery. Stem cell transplantation via both routes was previously found to result in the recovery of dystrophin expression, with differences in the levels restored in dystrophic mice [6, 9–12]. Intraarterial delivery can decrease the trapping of stem cells in filter organs, such as the lung, ultimately increasing stem cell distribution in the target tissue. As a result, intraarterial delivery has been used in multiple animal models and humans [11–14]. However, compared to intravenous injection, intraarterial injection is more challenging owing to the small size of and high blood pressure in the arteries of mice. Further, stem cells transported via intraarterial delivery can obstruct the microcirculation of muscles in mice [15, 16]. Thus, in the present study, we selected intravenous delivery as the systemic transplantation method. This method is not only safe but is also the most feasible and least invasive method [17, 18].

Although systemic transplantation is suitable for dystrophinopathy, the low efficacy makes its application challenging [6, 19]. The primary reason for insufficient efficacy is the limited stem cell homing toward injured muscles following systemic transplantation, which results in their extensive distribution in different tissues and organs [9]. Therefore, improved ADSC muscle homing is a key factor associated with effective systemic transplantation [20, 21].

The chemokine network is a complex system that mediates cell chemotaxis, with cells expressing chemokine receptors capable of migrating toward specific chemokines [22]. Chemokines released from the injured tissue can recruit cells expressing corresponding chemokine receptors, such as immune cells, via chemotaxis. Furthermore, stem cells can also respond to chemokines [20, 21], and increased stem cell homing can be achieved through increased chemokine release from injured tissue or expression of chemokine receptors on stem cells [23–25]. The latter strategy is advantageous for dystrophinopathy treatment, because it is difficult to increase chemokine release in every injured muscle.

The effect of increased expression of chemokine receptors on stem cell muscle homing as well as the chemokine receptor optimal for targeting in dystrophinopathy are yet unknown. Therefore, this study investigated chemokine expression profiles in muscles of patients with dystrophinopathy and dystrophic mice and determined a candidate chemokine receptor. We then verified whether the ADSC muscle homing and engraftment in a dystrophic mouse model was improved by targeting the candidate chemokine receptor.

## Materials and methods

### Animals

C57BL/10ScSn-Dmd^mdx^/J (mdx) mice, the most commonly used dystrophic mouse model, were originally purchased from Jackson Laboratory (Bar Harbor, ME, USA). C57BL/6-Tg (CAG-EGFP)C14-Y01-FM131Osb mice expressing green fluorescent protein (GFP) were purchased from the Biomedical Research Institute of Nanjing University (Nanjing, JS, China). C57BL/6 J (C57) mice were purchased from Guangdong Medical Laboratory Animal Center (Guangzhou, GD, China) and Beijing Vital River Laboratory Animal Technologies (Beijing, China). Mice were housed in a specific pathogen-free animal facility at Sun Yat-sen University and fed a standard diet and water under a 12-h/12-h light/dark cycle. The animal protocol was approved by the Animal Care and Experimentation Committee of Sun Yat-sen University.

### Bioinformatics analysis of microarray data

Microarray data were downloaded from the Gene Expression Omnibus (https://www.ncbi.nlm.nih.gov/geoprofiles/) using the search keyword “(DMD or BMD or dystrophinopathy or mdx) and CCL2.” The inclusion criteria of the microarray dataset were as follows: (1) data from muscle samples obtained from patients or mdx mice and (2) mouse data collected at 8 weeks of age (the age at which mdx mice undergo transplantation). The exclusion criteria were as follows: (1) datasets without raw data; (2) a sample size too low for statistical analysis; (3) use of special treatment conditions, such as a particular diet; and (4) if multiple gene chips were used in a dataset, chip data that could not be analysed individually were excluded.

Raw data were analysed using the ArrayAnalysis tool (http://www.arrayanalysis.org) [26]. Following background correction, data were normalised using GC-RMA method (quantile normalisation). Comparisons of gene expression between different groups were performed using the limma adapted *t* test, and *P* values were adjusted according to the false discovery rate. We then performed log_2_ transformation on the fold change, with positive values indicating that compared with controls, the expression of genes in the disease group was upregulated, whereas negative values indicated downregulation, and 0 indicated no difference.

Heatmaps were drawn using all log_2_(fold change) data, and bar graphs were drawn using log_2_(fold change) data with an adjusted *P* < 0.05. The Venn diagram included chemokines exhibiting differential expression (adjusted *P* < 0.05) in both human and mouse data. Strong evidence as chemokine candidates was determined according to similar significant differential expression in at least three datasets.

### Western blot

Mdx mice (8 weeks old) were sacrificed, and tissues and organs, including the tibialis anterior (TA), gastrocnemius, quadriceps, diaphragm, heart, liver, lung, kidney, stomach, intestine, brain, and spinal cord, were isolated. The contents in the stomach and intestine were removed. All tissues and organs were rapidly frozen in liquid nitrogen and used to prepare tissue lysate, which was extracted using radioimmunoprecipitation assay buffer (Beyotime, Shanghai, China) containing a protease inhibitor cocktail (Thermo Fisher Scientific, Waltham, MA, USA). For cell samples, membrane proteins were extracted from the cell lysate using the ProteoExtract transmembrane protein extraction kit (Millipore, Billerica, MA, USA) according to the manufacturer’s instructions.

Protein concentrations were measured using a BCA protein assay kit (Thermo Fisher Scientific). Proteins were separated using sodium dodecyl sulphate polyacrylamide gel (5% stacking gel and 10% or 12% resolving gel) electrophoresis and transferred to polyvinylidene difluoride membranes (Millipore). The membrane was blocked with 5% bovine serum albumin (BSA; Beyotime) for 1 h at room temperature (RT) and then incubated with one of the following primary antibodies overnight at 4 °C: rabbit anti-C–C chemokine ligand (CCL)2 (1:2000; Abcam, Cambridge, MA, USA), goat anti-CCL7 (0.1 μg/mL; R&D Systems, Minneapolis, MN, USA), rabbit anti-C–C chemokine receptor (CCR)2 (1:500; Abcam), rabbit anti-tubulin (1:1000; Abcam), and rabbit anti-sodium potassium ATPase (1:1000; Abcam). After washing, the membrane was incubated with one of the following secondary antibodies for 1 h at RT: horseradish peroxidase (HRP)-conjugated goat anti-rabbit IgG (1:3000; Cell Signaling Technology, Danvers, MA, USA) and HRP-conjugated donkey anti-goat IgG (1:3000; Abcam). The protein bands were visualised using enhanced chemiluminescence (Millipore), and quantitative analysis of band intensity was performed using ImageJ software (v.1.52e; National Institutes of Health, Bethesda, MD, USA).

### ADSC isolation and culture

GFP male mice (8–10 weeks old) were sacrificed and disinfected with 75% ethanol. Adipose tissue in bilateral groins was isolated, and vessels were removed. Adipose tissue was digested with 0.1% collagenase I (Invitrogen, Carlsbad, CA, USA) for 40 min at 37 °C with gentle agitation and stopped using ADSC complete medium [Dulbecco’s modified Eagle’s medium (DMEM)/F12 (Gibco, Gaithersburg, MD, USA) supplemented with 10% foetal bovine serum (FBS; Gibco), 1× GlutaMAX (Gibco), and 1× penicillin-streptomycin (Gibco)]. Cells were collected by centrifugation at 1100 rpm for 10 min and resuspended using ADSC complete medium. After filtering the cell resuspension with a 40-μm strainer (Corning, Corning, NY, USA), cells were seeded in T25 flasks and cultured at 37 °C in an incubator with 5% CO_2_. ADSCs at low passage (< 8) were used in this study.

### Lentivirus transduction

Vehicle lentivirus (plenti-EF1a-mCherry-P2A-Puro) and CCR2-overexpressing lentivirus (plenti-EF1a-mCherry-P2A-Puro-CMV-Ccr2) were purchased from OBiO Technology (Shanghai, China). Lentivirus at a multiplicity of infection of 60 and 6 μg/mL polybrene (OBiO Technology) was used for the 16-h lentiviral transduction, followed by screening ADSCs with 1.5 μg/mL puromycin (Sigma-Aldrich, St. Louis, MO, USA) for 72 h. According to different treatments, cells were classified into three groups: control ADSCs without lentivirus transduction (CTL-ADSCs), ADSCs transduced with vehicle lentivirus (VEH-ADSCs), and ADSCs transduced with CCR2-overexpressing lentivirus (CCR2-ADSCs).

### Stem cell differentiation

We used the MSC adipogenic differentiation medium kit (Cyagen, Santa Clara, CA, USA) for adipogenic induction of ADSCs according to the manufacturer’s instructions. Briefly, each cycle comprised a 72-h cell culture with medium A and a 24-h cell culture with medium B. After five cycles, Oil Red O staining was used to evaluate the formation of lipid droplets.

We used the MSC osteogenic differentiation medium kit (Cyagen) for osteogenic induction of ADSCs according to the manufacturer’s instructions. Briefly, after 3 to 4 weeks of cell culture with osteogenesis medium, we used Alizarin Red S staining to evaluate the formation of calcium nodules.

Myogenic differentiation was according to a previous protocol [10]. Briefly, ADSCs were cultured using myogenic induction medium [DMEM supplemented with 10% FBS, 0.5 μM BIO (Santa Cruz Biotechnology, Dallas, TX, USA), 20 μM forskolin (Santa Cruz Biotechnology), and 10 ng/ml fibroblast growth factor-basic (PeproTech, Rocky Hill, NJ, USA)] for 7 days and then replaced with ADSC culture supernatant for another 21 days. After differentiation, expression of the myogenic markers, myoblast determination protein 1 (MyoD) and myosin heavy chain (MyHC), was evaluated using immunofluorescence staining, on day 14 and day 28, respectively.

### Immunocytostaining

Cells were fixed with 4% paraformaldehyde (Sigma-Aldrich) for 15 min at RT, followed by blocking with 5% BSA containing 0.3% Triton (Sigma-Aldrich) for 1 h at RT. The cells were then incubated overnight at 4 °C with mouse anti-MyoD (1:50; Santa Cruz Biotechnology) or mouse anti-MyHC (1:75; Developmental Studies Hybridoma Bank, Iowa City, IA, USA). After washing, the cells were incubated for 1 h at RT with Alexa Fluor 647-conjugated goat anti-mouse IgG (1:1000; Cell Signaling Technology). Fluoroshield with 4′,6-diamidino-2-phenylindole (DAPI; Sigma-Aldrich) was used for nucleus staining, and immunoreactivity was visualised using a fluorescence microscope (IX71; Olympus, Tokyo, Japan).

### Quantitative polymerase chain reaction (qPCR)

Total RNA was extracted from cells using TRIzol reagent (Thermo Fisher Scientific) according to the manufacturer’s instructions, and cDNA was synthesised using PrimeScript RT master mix (TaKaRa, Shiga, Japan). qPCR was performed with SYBR Premix Ex Taq II (TaKaRa) and in a thermal cycler (Bio-Rad, Hercules, CA, USA). The forward and reverse primers were as follows: *Ccr2*, 5′-ATCCACGGCATACTATCAACATC-3′ and 5′-CAAGGCTCACCATCATCGTAG-3′; and *β-actin*, 5′-GGCTGTATTCCCCTCCATCG-3′ and 5′-CCAGTTGGTAACAATGCCATGT-3′.

### Cell migration assay

The polycarbonate membrane (8-μm pore size) in the Transwell plate (Corning) was coated for 1 h to 2 h at 37 °C with 0.1% gelatine (Sigma-Aldrich). ADSCs (2.5 × 10^5^) suspended in DMEM/F12 were then added to the upper chamber, and DMEM/F12 supplemented with 0.1% FBS and mouse CCL2 (PeproTech) was added to the lower chamber. We used three concentrations of CCL2 (50, 100, and 150 ng/mL), and the blank control chamber containing no CCL2 was used to calculate the migration index. Cells were incubated for 12 h to 24 h at 37 °C in an incubator with 5% CO_2_, after which ADSCs attached in the upper surface of the membrane were removed, and those migrated from the upper chamber and attached in the lower surface of the membrane were stained with DAPI for cell counting. The migration index was defined as follows: (number of migrated ADSCs toward CCL2-containing medium/number of migrated ADSCs toward medium without CCL2).

### Stem cell transplantation

We used an insulin syringe to inject 300 μL of phosphate-buffered saline (PBS; Hyclone; GE Healthcare, Provo, UT, USA) or 1 × 10^6^ ADSCs suspended in 300 μL PBS into the tail vein of an mdx mouse (injection time: over 3 min). The ADSC suspension was shaken before injection to prevent cell aggregation. According to different treatments, 8-week-old male mice were classified into four groups: (1) C57 mice without treatment, (2) mdx mice receiving PBS injection (mdx + PBS), (3) mdx mice receiving VEH-ADSC transplantation (mdx + VEH-ADSC), and (4) mdx mice receiving CCR2-ADSC transplantation (mdx + CCR2-ADSC). Mice were assessed at 1 month, 6 months, and 1 year after treatment, and there were five mice in each group at each time point.

### Assessment of mice after transplantation

The motor function of mice was assessed by the four-limb hanging test according to the standard protocol [27]. After the mice received saline cardiac perfusion, the TA, quadriceps, and heart were isolated and frozen for 30 s to 60 s in isopentane (Guangzhou Chemical Industry, Guangzhou, GD, China) pre-cooled by liquid nitrogen. The frozen muscles were placed into a cryostat at − 20 °C, and optimal cutting temperature compound (Sakura Finetek, Torrance, CA, USA) was used for muscle embedding. Serial sections (6–10-μm thick) were cut and fixed in cold acetone (Guangzhou Chemical Industry) for 10 min. The tissue sections then underwent immunohistostaining, haematoxylin and eosin (H&E) staining, and Sirius Red staining to assess stem cell homing efficacy and therapeutic effects.

### Immunohistostaining

Tissue sections were re-warmed for 30 min at RT and blocked with 5% BSA for 1 h at RT, followed by incubation overnight at 4 °C with one or two of the following primary antibodies: rabbit anti-dystrophin (1:200; Abcam), rabbit anti-laminin (1:200; Abcam), and chicken anti-GFP (1:1000; Abcam). After washing, the tissue sections were incubated for 1 h at RT with one or two of secondary antibodies: Alexa Fluor 350-conjugated goat anti-rabbit IgG (1:1000; GeneCopoeia, Rockville, MD, USA) and Alexa Fluor 488-conjugated goat anti-chicken IgG (1:1000; Invitrogen). Immunoreactivity was visualised using a fluorescence microscope (ECLIPSE Ni-U; Nikon, Tokyo, Japan).

### H&E staining

Tissue sections were processed according to previously described methods [28]. Briefly, tissue sections were stained using haematoxylin (Baso Diagnostic, Zhuhai, GD, China) for 3 min, incubated in 1% hydrochloric acid in ethanol (Guangzhou Chemical Industry) for 3 s, and then incubated with bluing reagent (Solarbio, Beijing, China) for 1 min. After rinsing, the sections were stained using eosin (Baso Diagnostic) for 30 s, followed by dehydration with ethanol (Guangzhou Chemical Industry) and clearance with xylene (Guangzhou Chemical Industry).

### Sirius Red staining

Tissue sections were stained using Sirius Red solution (Sigma-Aldrich) for 1 h. After rinsing, the sections were stained using haematoxylin (Sigma-Aldrich) for 8 min and rinsed for 10 min, followed by dehydration with ethanol (Guangzhou Chemical Industry) and clearance with xylene (Guangzhou Chemical Industry).

### Statistical analysis

Data were analysed using SPSS (v.20.0; IBM Corp., Armonk, NY, USA) and GraphPad (v.7.0; GraphPad Software, La Jolla, CA, USA). Data with a normal distribution were presented as the mean ± standard deviation. Student’s *t* test and analysis of variance were used for comparisons between two groups and multiple groups, respectively. Pairwise comparisons were performed using the least significant difference test. A *P* < 0.05 was considered significant. To analyse data from serial sections of muscles, the section with the largest GFP/dystrophin expression or the maximal percentage of myofibres with central nuclei was chosen as the mouse data used for statistical analysis.

## Results

### CCR2 identification as a potential target for improving stem cell muscle homing in dystrophinopathy

To determine a candidate chemokine axis, we evaluated four human microarray datasets (GSE465, GSE1004, GSE3307, and GSE6011) and six mouse microarray datasets (GSE1008, GSE1026, GSE1025, GSE897, GSE7187, and GSE1471). Additionally, we included three sub-datasets in GSE897 analysing extensor digitorum longus, quadriceps, and soleus, respectively, resulting in a total of eight mouse datasets (Table 1).

**Table 1:** The information of microarray datasets

Dataset	Gene chip	species	Muscle	Reference
GSE465	GPL91	Human	Unknown	Chen et al. [29]
GSE1004	GPL8300	Human	Quadriceps	Haslett et al. [30]
GSE3307	GPL96/GPL 97	Human	Quadriceps	Dadgar et al. [31]
GSE6011	GPL96	Human	Quadriceps	Pescatori et al. [32]
GSE1008	GPL81	Mouse	Extraocular muscle	Porter et al. [33, 34]
GSE1026	GPL81	Mouse	Diaphragm	Porter et al. [35]
GSE1025	GPL81	Mouse	Mixed samples of gastrocnemius and soleus	Porter et al. [33]
GSE897	GPL81	Mouse	Extensor digitorum longus/quadriceps /soleus	Haslett et al. [36]
GSE7187	GPL339	Mouse	Tibialis anterior	Baban et al. [37]
GSE1471	GPL339	Mouse	Cardiac muscle	Unpublished

**Fig. 1:**
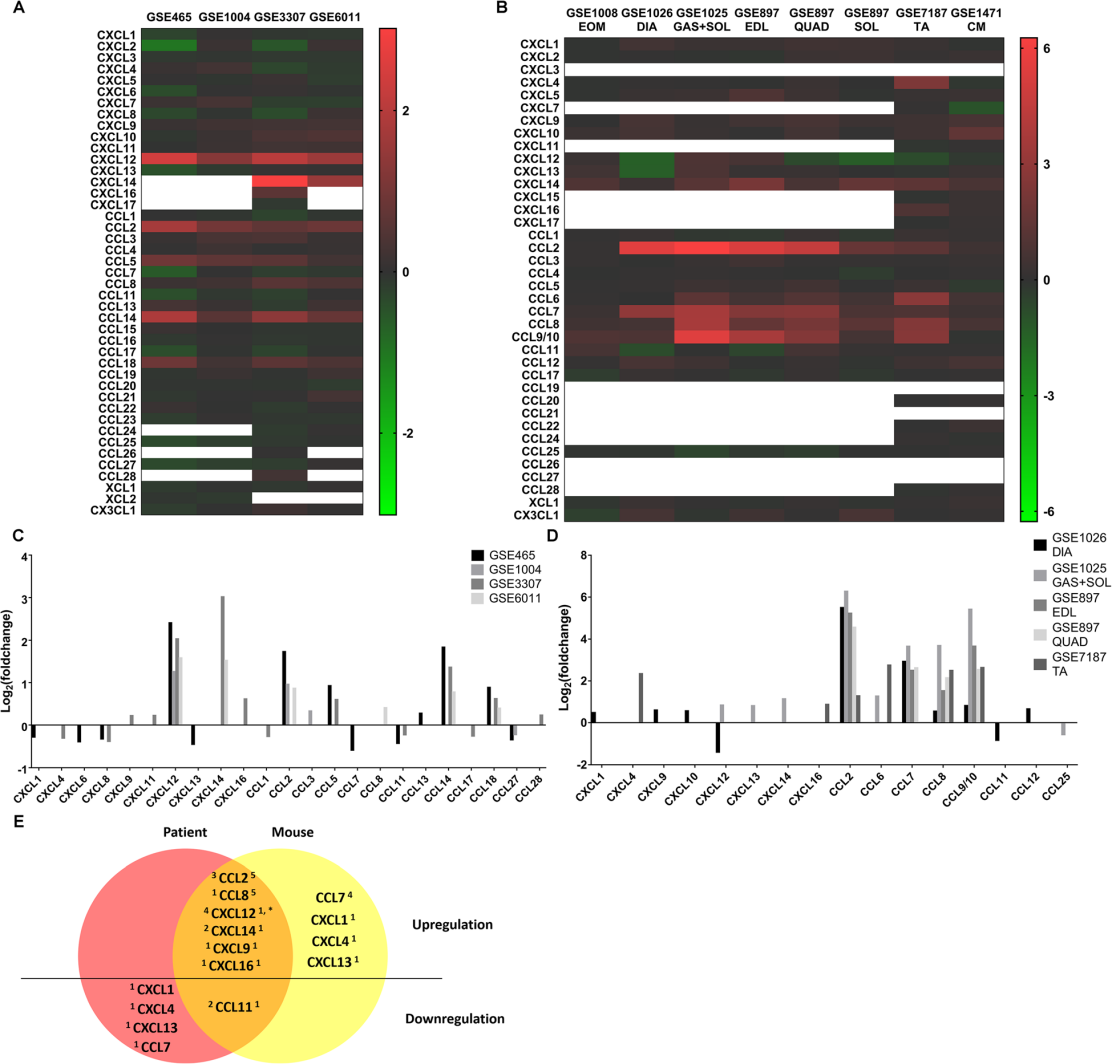
Microarray analysis of chemokine profiles in muscles of patients with dystrophinopathy and mdx mice. Heatmaps of differentially expressed chemokines in muscles of **a** patients with dystrophinopathy and **b** mdx mice relative to controls. Each row and column represents a chemokine and a dataset, respectively. Different colours indicate the log_2_(fold change) of each gene (red = upregulated, green = downregulated, and blank = no detection by the chip). Chemokines exhibiting significant differential expression in the muscles of **c** patients with dystrophinopathy and **d** mdx mice (adjusted *P* < 0.05). **e** Venn diagram of chemokines exhibiting significant differential expression in muscles of both patients and mdx mice. Red and yellow circles indicate the data from humans and mice, respectively. Orange overlapping regions indicate shared differential expression in both human and mouse data. Chemokines above and below the horizontal line are upregulated and downregulated, respectively. The superscript number next to the chemokine name indicates the number of datasets showing differential expression, with the superscripts on the left and right indicating human and mouse data, respectively. CXCL12^*^ showed contradictory results in two mouse datasets. EOM,extraocular muscle; DIA, diaphragm; GAS, gastrocnemius; SOL, soleus; EDL, extensor digitorum longus; QUAD, quadriceps; TA, tibialis anterior; CM, cardiac muscle

The results revealed differential chemokine expression in muscles from patients (Fig. 1a) and mdx mice (except for the extraocular muscle) (Fig. 1b) relative to that in controls. Additionally, we identified 23 chemokines exhibiting significant differential expression in at least one human dataset (Fig. 1c). Among these, four chemokines [C–X–C chemokine ligand (CXCL)12, CCL2, CCL14, and CCL18] showed upregulation in at least three human datasets. In mdx mice, there were three datasets with no significant differential expression of chemokines (GSE1008, GSE897 soleus, and GSE1471); however, among the remaining mdx datasets, we found 16 chemokines with significant differential expression in at least one mouse dataset (Fig. 1d). Among these, four chemokines (CCL2, CCL7, CCL8, and CCL9/10) showed upregulation in at least three datasets.

Given the clinical translation of the animal study, we analysed human and mouse data together (Fig. 1e). The results revealed six differentially upregulated chemokines (CCL2, CCL8, CXCL12, CXCL14, CXCL9, and CXCL16) in the muscles of both mdx mice and patients. Among these, CCL2 was the strongest candidate based on its upregulation in three human and five mouse datasets. Furthermore, the level of upregulation was relatively high, with fold changes ranging from 1.82 to 3.32 in the human datasets and 2.42 to 77.01 in the mouse datasets. Therefore, CCL2 and its receptor CCR2 were considered the candidate chemokine axis. Additionally, the other CCR2 ligand (CCL7) was significantly upregulated in the mouse datasets (fold change 1.84–12.57).

These findings identified the CCR2 ligands, CCL2 and CCL7, as being upregulated in injured muscles of mdx mice, suggesting that their levels in these injured muscles (as the primary injured tissue) might be higher than those in other organs and tissues.

### CCL2 and CCL7 levels in muscle tissue are higher than those in other tissues and organs in mdx mice

**Fig. 2:**
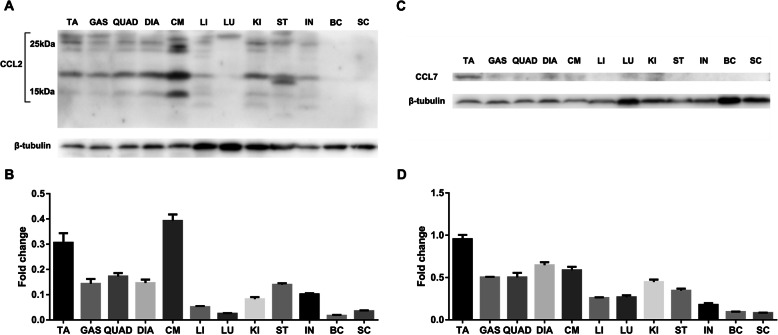
Protein levels of CCL2 and CCL7 in muscles are higher than those in other tissues and organs of mdx mice. Western blot results showing relative levels of CCL2 and CCL7 in different tissues and organs of mdx mice. **a** CCL2 expression and **b** levels relative to β-tubulin in different tissues and organs. **c** CCL7 expression and **d** levels relative to β-tubulin in different tissues and organs. TA, Tibialis anterior; GAS, gastrocnemius; QUAD, quadriceps; DIA, diaphragm; CM, cardiac muscle; LI, liver; LU, lung; KI, kidney; ST, stomach; IN, intestine; BC, brain cortex; SC, spinal cord

To determine whether CCL2 and CCL7 are mainly upregulated in the muscle tissue of mdx mice, we compared their protein levels in different tissues and organs of mdx mice, including the TA, gastrocnemius, quadriceps, diaphragm, cardiac muscle, liver, lung, kidney, stomach, intestine, brain cortex, and spinal cord. The results indicated that CCL2 (Fig. 2a, b) and CCL7 (Fig. 2c, d) levels in muscle tissue were higher than those in other tissues and organs. These results suggested that CCR2 as a potential candidate improving stem cell muscle homing due to the relatively high expression of its ligands in muscle tissue of mdx mice.

### Establishment of CCR2-overexpressing ADSCs

**Fig. 3:**
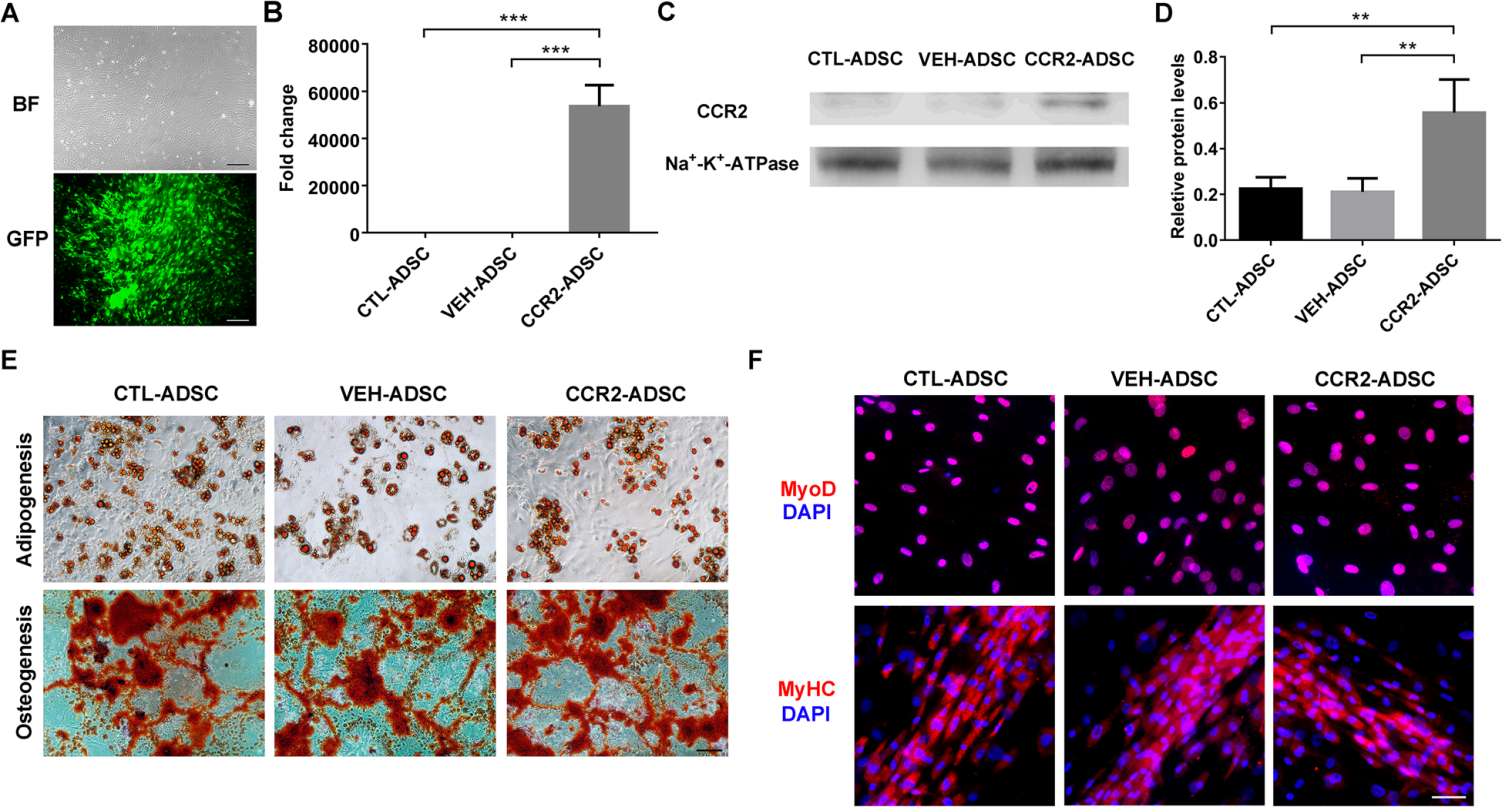
Establishment of CCR2-overexpressing ADSCs. **a** ADSC morphology and spontaneous green fluorescence. **b** *Ccr2* mRNA levels in CTL-ADSCs, VEH-ADSCs, and CCR2-ADSCs relative to CTL-ADSCs. **c** CCR2 protein expression and **d** levels relative to Na^+^-K^+^-ATPase in CTL-ADSCs, VEH-ADSCs, and CCR2-ADSCs. Na^+^-K^+^-ATPase was the reference protein for membrane protein samples. **e** Adipogenesis and osteogenesis of CTL-ADSCs, VEH-ADSCs, and CCR2-ADSCs according to the formation of lipid droplets and calcium nodules, respectively. **f** Myogenesis of CTL-ADSCs, VEH-ADSCs, and CCR2-ADSCs according to the expression of MyoD and MyHC. Scale bars **a** = 200 μm, **e** = 100 μm, and **f** = 50 μm. **P < 0.01, ***P < 0.001

We observed a short-spindle-like morphology of ADSCs cultured in vitro, and ADSCs isolated from GFP mice showed spontaneous green fluorescence (Fig. 3a). Following lentiviral transduction, we confirmed significant elevations in *Ccr2* mRNA and CCR2 protein levels in CCR2-ADSCs relative to the levels in CTL-ADSCs and VEH-ADSCs (Fig. 3b–d), which indicated the successful establishment of CCR2-overexpressing ADSCs.

Assessment of cell differentiation indicated that CTL-ADSCs, VEH-ADSCs, and CCR2-ADSCs were able to form lipid droplets and calcium nodules following adipogenic and osteogenic induction, respectively (Fig. 3e). Moreover, the number of lipid droplets and calcium nodules did not differ between groups. Furthermore, following myogenic induction, all three groups expressed MyoD and MyHC, with no differences observed between groups (Fig. 3e). These results indicated that CCR2 overexpression did not affect the multilineage-differentiation potentials of the ADSCs.

### CCR2 improves ADSC migration

**Fig. 4:**
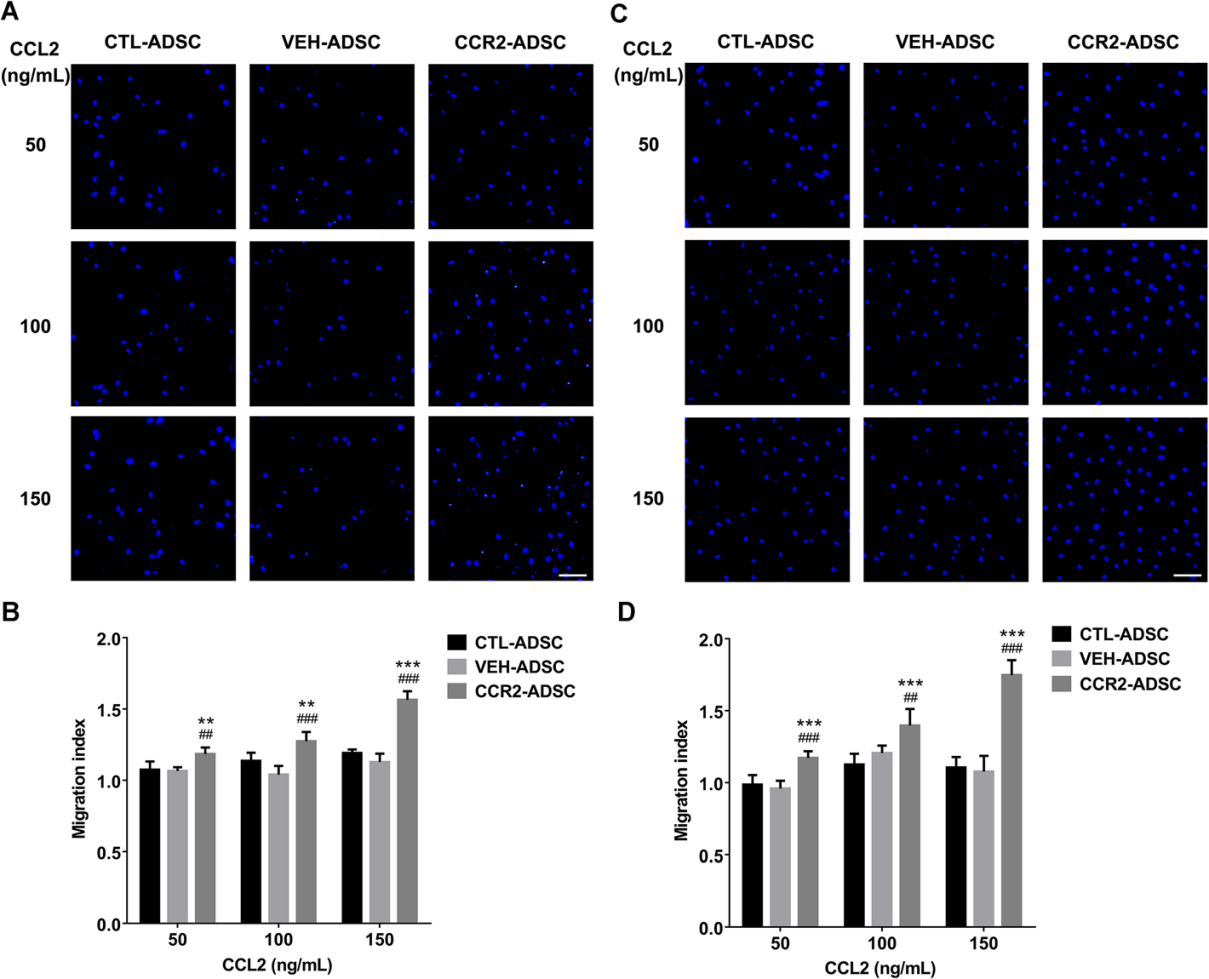
CCR2 improves ADSC migration. Three CCL2 concentrations were evaluated (50, 100, and 150 ng/mL), and DAPI staining of nuclei (blue) was used to calculate the number of migrated ADSCs. **a** DAPI staining of migrated ADSCs and **b** migration index for CTL-ADSCs, VEH-ADSCs, and CCR2-ADSCs after 12 h. **c** DAPI staining of migrated ADSCs and **d** migration index for CTL-ADSCs, VEH-ADSCs, and CCR2-ADSCs after 24 h. Scale bar = 100 μm. **P < 0.01, ***P < 0.001, vs. CTL-ADSC; ^##^*P* < 0.01, ^###^*P* < 0.001 vs. VEH-ADSC

To determine whether CCR2 overexpression affects cell migration, we compared the migratory ability of CTL-ADSCs, VEH-ADSCs, and CCR2-ADSCs. The results indicated that CCR2-ADSCs exhibited a higher migration index relative to CTL-ADSCs and VEH-ADSCs after a 12-h incubation (Fig. 4a, b). After 24 h, differences in migration between CCR2-ADSCs and other groups became more pronounced (Fig. 4c, d). Furthermore, we found that migration of the CCR2-ADSCs was CCL2-concentration-dependent and increased along with CCL2 levels. Given that we observed the same biological characteristics between CTL-ADSCs and VEH-ADSCs, we chose VEH-ADSCs as control cells for the in vivo experiments.

### CCR2-ADSC transplantation further improves the motor function of mdx mice

**Fig. 5:**
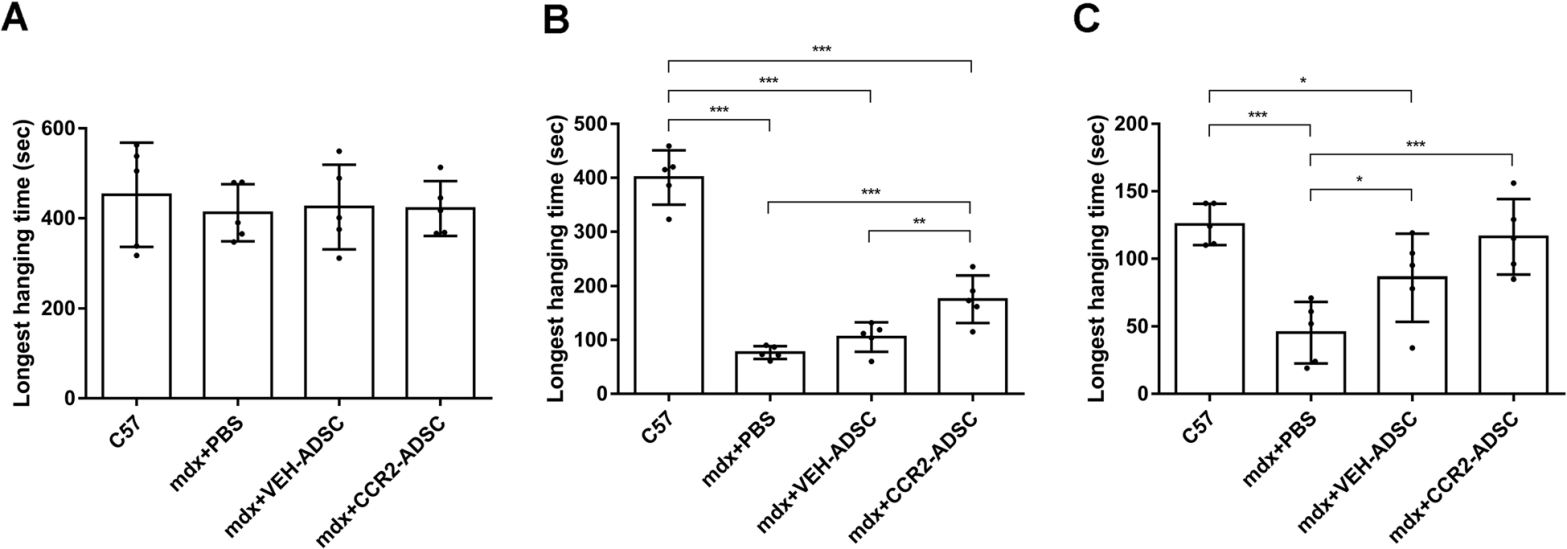
CCR2-ADSC transplantation further improves the motor function of mdx mice. The four-limb hanging test was used to evaluate motor function in mice. The longest hanging time for C57, mdx + PBS, mdx + VEH-ADSC, and mdx + CCR2-ADSC mice at **a** 1-month, **b** 6-month, and **c** 1-year post-transplantation of ADSCs. *P < 0.05, **P < 0.01, ***P < 0.001

The recovery of motor function in mdx mice is among the most important evidence of positive therapeutic effects [27]. Here, we used the four-limb hanging test to evaluate motor function, with the results indicating no difference in the longest hanging time between C57, mdx + PBS, mdx + VEH-ADSC, and mdx + CCR2-ADSC mice at 1-month post-transplantation (Fig. 5a). This was suggestive of the mild phenotype in the mdx mouse model, the motor function of which does not deteriorate in the early stage. However, at 6 months post-transplantation, the motor function of mdx + PBS mice decreased significantly, and the longest hanging time of mdx + CCR2-ADSC mice significantly increased relative to that of mdx + PBS and mdx + VEH-ADSC mice (Fig. 5b). At 1-year post-transplantation, the longest hanging time of mdx + CCR2-ADSC mice remained significantly higher than that of mdx + PBS mice. Moreover, compared with mdx + VEH-ADSC mice, we observed a trend of increased motor function in mdx + CCR2-ADSC mice, although the difference was insignificant (Fig. 5c). These results demonstrated a better therapeutic effect from CCR2-ADSC transplantation than that from VEH-ADSC transplantation.

### CCR2 promotes muscle homing of ADSCs in mdx mice

**Fig. 6:**
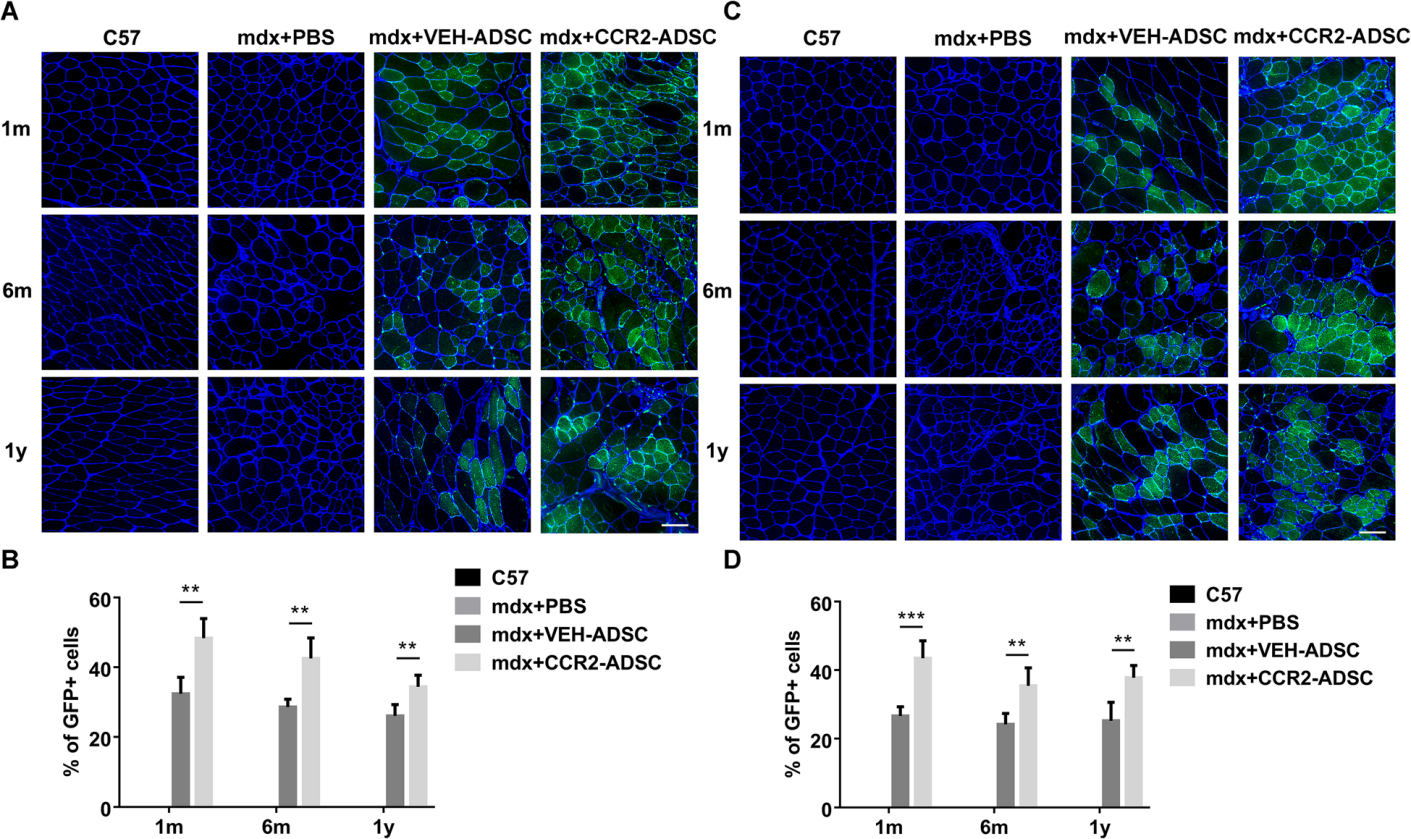
CCR2 promotes muscle homing of ADSCs in mdx mice. Green fluorescent protein (GFP)-positive cells were used to evaluate muscle homing of ADSCs. Green: GFP; blue: laminin (indicating the basement membrane). **a** GFP-positive cells and **b** their percentages in the tibialis anterior of C57, mdx + PBS, mdx + VEH-ADSC, and mdx + CCR2-ADSC mice at 1-month, 6-month, and 1-year post-transplantation of ADSCs. **c** GFP-positive cells and **d** their percentages in the quadriceps of C57, mdx + PBS, mdx + VEH-ADSC, and mdx + CCR2-ADSC mice at 1-month, 6-month, and 1-year post-transplantation of ADSCs. Scale bar = 100 μm. **P < 0.01, ***P < 0.001

GFP-positive cells in the muscles of transplanted mice allowed evaluation of the muscle-homing efficacy of the GFP-expressing donor ADSCs. Here, we chose two separate hindlimb muscles (TA and quadriceps) to provide additional evidence. We did not observe GFP expression in C57 or mdx + PBS mice; however, GFP-positive myofibres were observed in the TA of mdx + VEH-ADSC mice. Furthermore, these GFP-positive cells continued to survive up to 1-year post-transplantation (Fig. 6a). Notably, the percentage of GFP-positive myofibres in mdx + CCR2-ADSC mice was higher than that in mdx + VEH-ADSC mice at 1-month, 6-month, and 1-year post-transplantation (Fig. 6a, b). Similarly, this difference in percentage between mdx + VEH-ADSC and mdx + CCR2-ADSC mice was also observed in the quadriceps (Fig. 6c, d). These results indicated that CCR2 promoted muscle homing of ADSCs in mdx mice.

We did not observe the presence of GFP-positive cells in the hearts of mdx + VEH-ADSC and mdx + CCR2-ADSC mice (data not shown), which aligned with previous results [7, 10, 12]. This result may be due to the limited survival and differentiation ability of ADSCs in cardiac tissue [38].

### CCR2-ADSCs further restores dystrophin expression

**Fig. 7:**
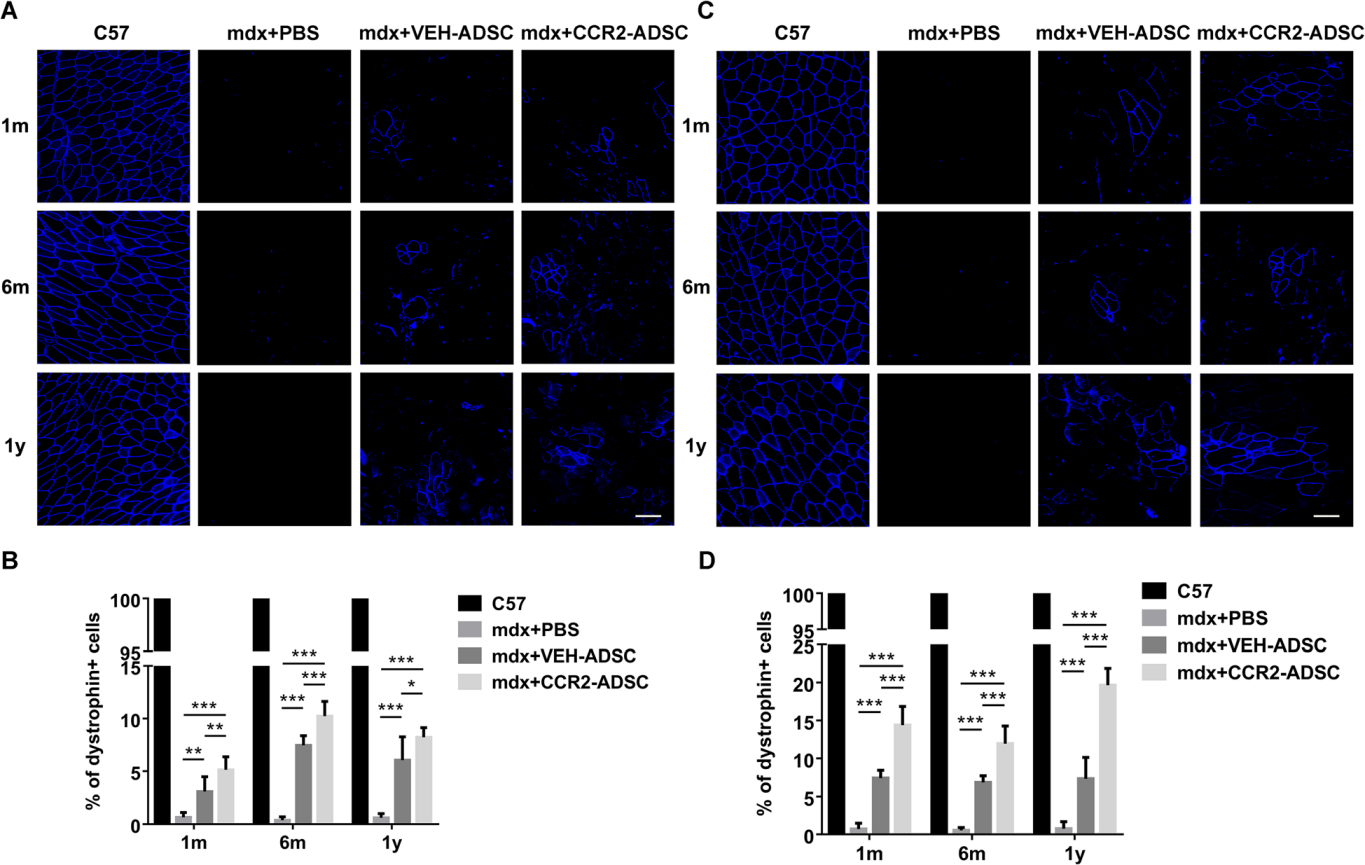
CCR2-ADSC transplantation further improves dystrophin expression in muscles of mdx mice. **a** Dystrophin expression and **b** the percentages of dystrophin-positive cells in the tibialis anterior of C57, mdx + PBS, mdx + VEH-ADSC, and mdx + CCR2-ADSC mice at 1-month, 6-month, and 1-year post-transplantation of ADSC. Blue: dystrophin. **c** Dystrophin expression and **d** the percentages of dystrophin-positive cells in the quadriceps of C57, mdx + PBS, mdx + VEH-ADSC, and mdx + CCR2-ADSC mice at 1-month, 6-month, and 1-year post-transplantation of ADSC. Scale bar = 100 μm. *P < 0.05, **P < 0.01, ***P < 0.001

Dystrophin expression represents an important biomarker for evaluating the efficacy of stem cell transplantation in dystrophinopathy. Our results showed that dystrophin was expressed evenly in the sarcolemma of C57 mice but at very low levels in the muscle of mdx + PBS mice (Fig. 7a–d), which was consistent with the percentage of revertant fibres in mdx mice (< 1%) [8, 39, 40]. Partial recovery of dystrophin expression in mdx + VEH-ADSC mice at 1-month, 6-month, and 1-year post-transplantation indicated the long-term benefit of ADSC transplantation. Notably, we observed a significant increase in dystrophin expression in the TA (Fig. 7a, b) and quadriceps (Fig. 7c, d) of mdx + CCR2-ADSC mice relative to mdx + VEH-ADSC mice at all post-transplantation time points. These findings showed that CCR2-ADSC transplantation further restored dystrophin expression in mdx mice.

### CCR2-ADSCs enhance protection of muscles from injury

**Fig. 8:**
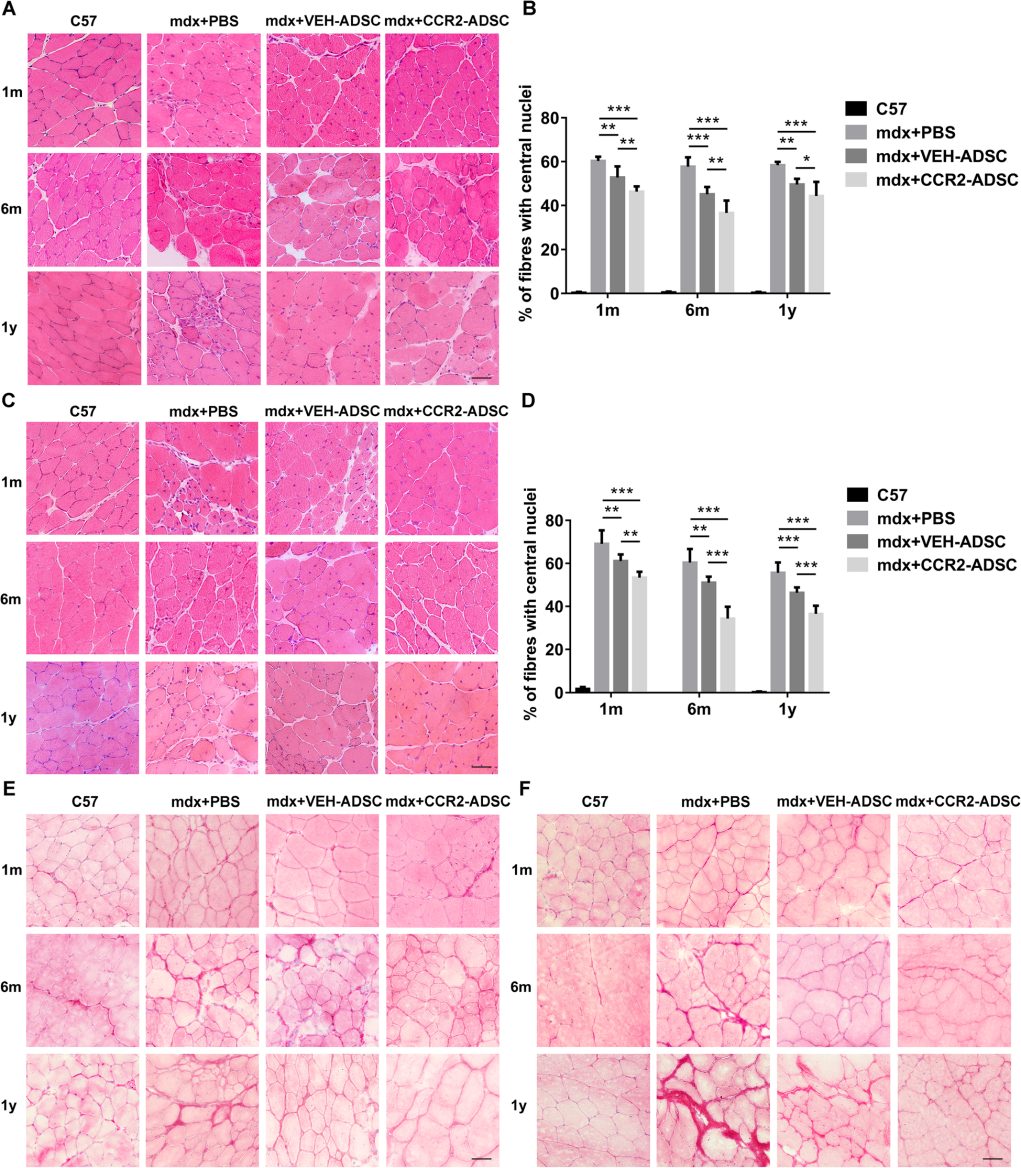
CCR2-ADSC transplantation further improves muscle pathology in mdx mice. Assessment of muscle pathology using haematoxylin and eosin (H&E) and Sirius Red staining. The decreased percentage of myofibres with central nuclei correlated with the decreased severity of muscle injury in mdx mice. Moreover, muscle fibrosis was assessed using Sirius Red staining, which revealed collagen expression. The decreased collagen expression levels correlated with the decreased severity of muscle fibrosis in mdx mice. **a** H&E-stained images and **b** the percentages of myofibres with central nuclei in the tibialis anterior (TA) of C57, mdx + PBS, mdx + VEH-ADSC, and mdx + CCR2-ADSC mice at 1-month, 6-month, and 1-year post-transplantation of ADSCs. **c** H&E-stained images and **d** the percentages of myofibres with central nuclei in the quadriceps of C57, mdx + PBS, mdx + VEH-ADSC, and mdx + CCR2-ADSC at 1-month, 6-month, and 1-year post-transplantation of ADSCs. Fibrosis in the TA (**e**) and quadriceps (**f**) of C57, mdx + PBS, mdx + VEH-ADSC, and mdx + CCR2-ADSC mice at 1-month, 6-month, and 1-year post-transplantation of ADSCs. Scale bar = 50 μm. *P < 0.05, **P < 0.01, ***P < 0.001

In normal muscle, nuclei are distributed under the sarcolemma; however, nuclei translocate toward the centre of myofibres in mdx mice, which indicates the occurrence of muscle injury [41]. Therefore, the percentage of myofibres with central nuclei can denote the severity of muscle injury in dystrophinopathy. In the TA, we found that the percentage of myofibres with central nuclei significantly decreased in mdx + VEH-ADSC mice relative to that in mdx + PBS mice at 1-month, 6-month, and 1-year post-transplantation (Fig. 8a, b), indicating long-term protection following ADSC transplantation. Notably, these percentages further decreased in mdx + CCR2-ADSCs at each post-transplantation time point (Fig. 8a, b). Furthermore, we observed similar results of CCR2-ADSC transplantation in the quadriceps of mdx mice (Fig. 8c, d).

Furthermore, fibrosis is an important pathological alteration in dystrophic muscles, and also an indicator of muscle injury [2]. A decrease in muscle fibrosis was revealed in mdx + CCR2-ADSC mice when compared with mdx + VEH-ADSC mice, and this decrease was more obvious in older mice (Fig. 8e, f). These results demonstrated that transplantation of CCR2-ADSCs further protected muscles from injury in mdx mice.

## Discussion

Dystrophinopathy currently lacks a cure. Systemic transplantation of ADSCs has a therapeutic effect on dystrophic mice and improving stem cell muscle homing is important for increasing efficacy [6, 20, 21]. In this study, we identified chemokine profiles exhibiting significant alterations in injured muscles from patients and mdx mice relative to that in controls. Additionally, we identified CCR2 ligands differentially upregulated in injured muscles of mdx mice. Furthermore, we found that CCR2 overexpression improved ADSC migration and muscle homing and enhanced post-transplantation therapeutic effects in mdx mice through further restoration of dystrophin expression and protection from injury.

The changes in chemokine profile in the muscles of patients and mdx mice were associated with muscle injury, which explains their absence in extraocular muscle [33]. It is possible to exploit these altered chemokines to enhance the homing ability of stem cells toward target lesions (injured muscles) via modification of corresponding chemokine receptors. Although microarray analysis of chemokine expression is helpful in determining candidate chemokine receptors, limitations exist that can hinder interpretation. In this study, we found inconsistencies in differences between chemokine expression in the quadriceps and TA of mdx mice according to microarray and Western blotting results (Figs. 1 and 2), likely due to inaccuracies in the calculated fold changes during microarray analysis [42]. Therefore, validation of microarray analysis using other traditional methods is recommended prior to determining a candidate chemokine receptor.

The choice of a candidate chemokine receptor for improved stem cell homing would depend on the chemokine levels in the lesion, strength of evidence, chemokine distribution in tissues and organs, and findings of previous studies. In this study, we chose CCR2 based on the following findings: (1) strong evidence associated with significant upregulation of its ligands; (2) elevated expression of both CCL2 and CCL7 in injured muscles of mdx mice relative to other tissues and organs; (3) published results demonstrating a role for CCL2–CCR2 in the chemotaxis of monocytes/macrocytes, which caused their accumulation in injured muscles in patients with dystrophinopathy and dystrophic mice [43, 44]; and (4) previous studies reporting participation of the CCL2–CCR2 axis in MSC homing toward injured heart and infarcted brain [24, 45]. Integrated analysis is important for identifying optimal candidate chemokine receptors; however, use of this strategy to improve stem cell engraftment in other diseases requires re-evaluation of differential chemokine expression according to the conditions of the targeted disease. Nevertheless, our findings suggest that the strategy used in the present study can be considered effective for other diseases.

CCR2 overexpression did not affect the adipogenic and osteogenic potentials of mouse ADSCs, which agreed with the results of a previous study using human bone marrow-derived MSCs [24]. In terms of myogenic potential, a previous study reported that *Ccr2* knockout decreased muscle regeneration in mice [46]. In the present study, we showed that CCR2 overexpression did not enhance the MyoD and MyHC levels following myogenic differentiation of ADSCs, suggesting that CCR2 might not play an important role in myogenic differentiation. Furthermore, *Ccr2* knockout could lead to numerous changes in the muscle microenvironment important for muscle regeneration [47, 48]; therefore, the role of CCR2 in muscle regeneration might be mainly associated with the microenvironment rather than the biological characteristics of stem cells.

Previous studies have reported the therapeutic effects of ADSC systemic transplantation in mdx mice [6], and the results in the present study were consistent with those findings and confirmed the long-term protective effects of ADSC systemic transplantation. This therapeutic effect is associated with the restoration of dystrophin following ADSC-mediated muscle regeneration. Moreover, a correlation between increased dystrophin expression and decreased phenotype severity has been widely reported [2, 3, 49] and was confirmed in the present study in mdx-VEH-ADSC and mdx-CCR2-ADSC mice.

Furthermore, our results indicated that CCR2 improved ADSC-transplantation efficacy. The further recovery of motor function in mdx + CCR2-ADSC mice resulted from further recovery of dystrophin expression and protection against muscle injury through the improved homing of ADSCs, which resulted in a higher number of donor cells in injured muscles. Moreover, the improved homing ability was a product of enhanced migration mediated by CCR2 overexpression. CCR2 binding by ligands released from injured muscles initiates CCR2-mediated cell-migratory processes, including CCR2 polarisation, signal transduction, actin reconstruction, and directional cell movement toward regions with high ligand concentrations [45, 50, 51]. These characteristics make CCR2 an important promoter of directional migration, with this role confirmed in both previous studies [24, 45, 50, 51] and the present work. However, we observed that in vitro CCR2 levels in ADSCs remained relatively low associated with the gradual loss of surface proteins by MSCs during in vitro culture [52]. Therefore, increasing CCR2 expression is an important approach to enhance CCR2-mediated migration and homing of ADSCs, and establishment of a safe and effective method to upregulate CCR2 levels in ADSCs will accelerate clinical translation of this method.

The method used to evaluate the number of donor ADSCs in muscles can be used to compare the relative number of donor ADSCs but not calculate the absolute number of homed ADSCs, because myofibres harbour multiple nuclei, and those containing at least one donor nucleus could present green fluorescence in the cytoplasm. Additionally, some treatments target the CCL2–CCR2 axis to improve muscle pathology in dystrophinopathy by decreasing the accumulation of monocytes/macrocytes [43, 44, 53], which can potentially affect the homing of CCR2-ADSCs. Our results indicated a large number of ADSCs homing to injured muscles at 1-month post-transplantation, with no obvious elevation of this number at 6-month and 1-year post-transplantation. Additionally, a previous study reported that signalling by donor MSCs stabilised 1 month after their systemic transplantation in mdx mice [9]. These findings suggest that a 1-month interval following cell transplantation is sufficient when these two treatments are combined.

## Conclusion

CCR2 improved muscle homing and the therapeutic effects of ADSC systemic transplantation in dystrophic mice, highlighting the importance of homing efficacy in stem cell transplantation. These results suggested that the use of the chemokine system represents an efficacious strategy for improving stem cell homing.

## Data Availability

The datasets analysed during the current study are available in the Gene Expression Omnibus repository (https://www.ncbi.nlm.nih.gov/geoprofiles/) under accession number GSE465 [29], GSE1004 [30], GSE3307 [31], GSE6011 [32], GSE1008 [33, 34], GSE 1026 [35], GSE 1025 [33], GSE897 [36], GSE7187 [37], and GSE1471 (unpublished).

## Abbreviations

ADSCs: Adipose-derived stem cells
CCR: C–C chemokine receptor
CCL: C–C chemokine ligand
DMD: Duchenne muscular dystrophy
BMD: Becker muscular dystrophy
MSC: Mesenchymal stem cell
GFP: Green fluorescent protein
TA: Tibialis anterior
BSA: Bovine serum albumin
RT: Room temperature
HRP: Horseradish peroxidase
DMEM: Dulbecco’s modified Eagle’s medium
FBS: Foetal bovine serum
CTL-ADSCs: Control ADSCs without lentivirus transduction
VEH-ADSCs: ADSCs transduced with vehicle lentivirus
CCR2-ADSCs: ADSCs transduced with CCR2-overexpressing lentivirus
MyoD: Myoblast determination protein 1
MyHC: Myosin heavy chain
DAPI: 4′,6-diamidino-2-phenylindole
qPCR: Quantitative polymerase chain reaction
Mdx + PBS: Mdx mice receiving PBS injection
Mdx + VEH-ADSC: Mdx mice receiving VEH-ADSC transplantation
Mdx + CCR2-ADSC: Mdx mice receiving CCR2-ADSC transplantation
H&E: Haematoxylin and eosin
CXCL: C–X–C chemokine ligand
EOM: Extraocular muscle
DIA: Diaphragm
GAS: Gastrocnemius
SOL: Soleus
EDL: Extensor digitorum longus
QUAD: Quadriceps
CM: Cardiac muscle
LI: Liver
KI: Kidney
ST: Stomach
IN: Intestine
BC: Brain cortex
SC: Spinal cord

## References

[1] Fox H, Millington L, Mahabeer I, van Ruiten H (2020). Duchenne muscular dystrophy. BMJ.

[2] Guiraud S, Aartsma-Rus A, Vieira NM, Davies KE, van Ommen GJ, Kunkel LM (2015). The pathogenesis and therapy of muscular dystrophies. Annu Rev Genomics Hum Genet.

[3] Verhaart IEC, Aartsma-Rus A (2019). Therapeutic developments for Duchenne muscular dystrophy. Nat Rev Neurol.

[4] Bajek A, Gurtowska N, Olkowska J, Kazmierski L, Maj M, Drewa T (2016). Adipose-derived stem cells as a tool in cell-based therapies. Arch Immunol Ther Exp.

[5] Lee EM, Kim AY, Lee EJ, Park JK, Lee MM, Hwang M (2015). Therapeutic effects of mouse adipose-derived stem cells and losartan in the skeletal muscle of injured mdx mice. Cell Transplant.

[6] Geng J, Liu G, Peng F, Yang L, Cao J, Li Q (2012). Decorin promotes myogenic differentiation and mdx mice therapeutic effects after transplantation of rat adipose-derived stem cells. Cytotherapy..

[7] Liu Y, Yan X, Sun Z, Chen B, Han Q, Li J (2007). Flk-1+ adipose-derived mesenchymal stem cells differentiate into skeletal muscle satellite cells and ameliorate muscular dystrophy in mdx mice. Stem Cells Dev.

[8] Rodriguez AM, Pisani D, Dechesne CA, Turc-Carel C, Kurzenne JY, Wdziekonski B (2005). Transplantation of a multipotent cell population from human adipose tissue induces dystrophin expression in the immunocompetent mdx mouse. J Exp Med.

[9] Feng SW, Lu XL, Liu ZS, Zhang YN, Liu TY, Li JL (2008). Dynamic distribution of bone marrow-derived mesenchymal stromal cells and change of pathology after infusing into mdx mice. Cytotherapy..

[10] Zhang Y, Zhu Y, Li Y, Cao J, Zhang H, Chen M (2015). Long-term engraftment of myogenic progenitors from adipose-derived stem cells and muscle regeneration in dystrophic mice. Hum Mol Genet.

[11] Matthias N, Hunt SD, Wu J, Darabi R (2015). Skeletal muscle perfusion and stem cell delivery in muscle disorders using intra-femoral artery canulation in mice. Exp Cell Res.

[12] Torrente Y, Tremblay JP, Pisati F, Belicchi M, Rossi B, Sironi M (2001). Intraarterial injection of muscle-derived CD34(+)Sca-1(+) stem cells restores dystrophin in mdx mice. J Cell Biol.

[13] Rouger K, Larcher T, Dubreil L, Deschamps JY, Le Guiner C, Jouvion G (2011). Systemic delivery of allogenic muscle stem cells induces long-term muscle repair and clinical efficacy in duchenne muscular dystrophy dogs. Am J Pathol.

[14] Dai A, Baspinar O, Yeşilyurt A, Sun E, Aydemir Çİ, Öztel ON (2018). Efficacy of stem cell therapy in ambulatory and nonambulatory children with Duchenne muscular dystrophy - phase I-II. Degener Neurol Neuromuscul Dis.

[15] Berry SE (2015). Concise review: mesoangioblast and mesenchymal stem cell therapy for muscular dystrophy: progress, challenges, and future directions. Stem Cells Transl Med.

[16] Furlani D, Ugurlucan M, Ong L, Bieback K, Pittermann E, Westien I (2009). Is the intravascular administration of mesenchymal stem cells safe? Mesenchymal stem cells and intravital microscopy. Microvasc Res.

[17] Ra JC, Shin IS, Kim SH, Kang SK, Kang BC, Lee HY (2011). Safety of intravenous infusion of human adipose tissue-derived mesenchymal stem cells in animals and humans. Stem Cells Dev.

[18] Misra V, Ritchie MM, Stone LL, Low WC, Janardhan V (2012). Stem cell therapy in ischemic stroke: role of IV and intra-arterial therapy. Neurology..

[19] Gang EJ, Darabi R, Bosnakovski D, Xu Z, Kamm KE, Kyba M (2009). Engraftment of mesenchymal stem cells into dystrophin-deficient mice is not accompanied by functional recovery. Exp Cell Res.

[20] Karp JM, Leng Teo GS (2009). Mesenchymal stem cell homing: the devil is in the details. Cell Stem Cell.

[21] Lapidot T, Dar A, Kollet O (2005). How do stem cells find their way home?. Blood..

[22] Kufareva I, Salanga CL, Handel TM (2015). Chemokine and chemokine receptor structure and interactions: implications for therapeutic strategies. Immunol Cell Biol.

[23] Li L, Wu S, Liu Z, Zhuo Z, Tan K, Xia H (2015). Ultrasound-targeted microbubble destruction improves the migration and homing of mesenchymal stem cells after myocardial infarction by upregulating SDF-1/CXCR4: a pilot study. Stem Cells Int.

[24] Huang Y, Wang J, Cai J, Qiu Y, Zheng H, Lai X (2018). Targeted homing of CCR2-overexpressing mesenchymal stromal cells to ischemic brain enhances post-stroke recovery partially through PRDX4-mediated blood-brain barrier preservation. Theranostics..

[25] Yang JX, Zhang N, Wang HW, Gao P, Yang QP, Wen QP (2015). CXCR4 receptor overexpression in mesenchymal stem cells facilitates treatment of acute lung injury in rats. J Biol Chem.

[26] Eijssen LM, Jaillard M, Adriaens ME, Gaj S, de Groot PJ, Müller M (2013). User-friendly solutions for microarray quality control and pre-processing on ArrayAnalysis.org. Nucleic Acids Res.

[27] Aartsma-Rus A, van Putten M (2014). Assessing functional performance in the mdx mouse model. J Vis Exp.

[28] Wang L, Chen M, Xu M, Li J, Feng P, He R, et al. Ratio of creatine kinase to alanine aminotransferase as a biomarker of acute liver injury in dystrophinopathy. Dis Markers. 2018;2018:6484610. 10.1155/2018/6484610 PMC6029496 30018675

[29] Chen YW, Zhao P, Borup R, Hoffman EP (2000). Expression profiling in the muscular dystrophies: identification of novel aspects of molecular pathophysiology. J Cell Biol.

[30] Haslett JN, Sanoudou D, Kho AT, Bennett RR, Greenberg SA, Kohane IS (2002). Gene expression comparison of biopsies from Duchenne muscular dystrophy (DMD) and normal skeletal muscle. Proc Natl Acad Sci U S A.

[31] Dadgar S, Wang Z, Johnston H, Kesari A, Nagaraju K, Chen YW (2014). Asynchronous remodeling is a driver of failed regeneration in Duchenne muscular dystrophy. J Cell Biol.

[32] Pescatori M, Broccolini A, Minetti C, Bertini E, Bruno C, D'amico A (2007). Gene expression profiling in the early phases of DMD: a constant molecular signature characterizes DMD muscle from early postnatal life throughout disease progression. FASEB J.

[33] Porter JD, Merriam AP, Leahy P, Gong B, Khanna S (2003). Dissection of temporal gene expression signatures of affected and spared muscle groups in dystrophin-deficient (mdx) mice. Hum Mol Genet.

[34] Porter JD, Merriam AP, Khanna S, Andrade FH, Richmonds CR, Leahy P (2003). Constitutive properties, not molecular adaptations, mediate extraocular muscle sparing in dystrophic mdx mice. FASEB J.

[35] Porter JD, Merriam AP, Leahy P, Gong B, Feuerman J, Cheng G (2004). Temporal gene expression profiling of dystrophin-deficient (mdx) mouse diaphragm identifies conserved and muscle group-specific mechanisms in the pathogenesis of muscular dystrophy. Hum Mol Genet.

[36] Haslett JN, Kang PB, Han M, Kho AT, Sanoudou D, Volinski JM (2005). The influence of muscle type and dystrophin deficiency on murine expression profiles. Mamm Genome.

[37] Baban D, Davies KE (2008). Microarray analysis of mdx mice expressing high levels of utrophin: therapeutic implications for dystrophin deficiency. Neuromuscul Disord.

[38] Yang D, Wang W, Li L, Peng Y, Chen P, Huang H (2013). The relative contribution of paracine effect versus direct differentiation on adipose-derived stem cell transplantation mediated cardiac repair. PLoS One.

[39] Arechavala-Gomeza V, Kinali M, Feng L, Guglieri M, Edge G, Main M (2010). Revertant fibres and dystrophin traces in Duchenne muscular dystrophy: implication for clinical trials. Neuromuscul Disord.

[40] Hoffman EP, Morgan JE, Watkins SC, Partridge TA (1990). Somatic reversion/suppression of the mouse mdx phenotype in vivo. J Neurol Sci.

[41] Briguet A, Courdier-Fruh I, Foster M, Meier T, Magyar JP (2004). Histological parameters for the quantitative assessment of muscular dystrophy in the mdx-mouse. Neuromuscul Disord.

[42] Kothapalli R, Yoder SJ, Mane S, Loughran TP (2002). Microarray results: how accurate are they?. BMC Bioinformatics.

[43] Mojumdar K, Liang F, Giordano C, Lemaire C, Danialou G, Okazaki T (2014). Inflammatory monocytes promote progression of Duchenne muscular dystrophy and can be therapeutically targeted via CCR2. EMBO Mol Med.

[44] Zhao W, Wang X, Ransohoff RM, Zhou L (2017). CCR2 deficiency does not provide sustained improvement of muscular dystrophy in mdx5cv mice. FASEB J.

[45] Belema-Bedada F, Uchida S, Martire A, Kostin S, Braun T (2008). Efficient homing of multipotent adult mesenchymal stem cells depends on FROUNT-mediated clustering of CCR2. Cell Stem Cell.

[46] Warren GL, Hulderman T, Mishra D, Gao X, Millecchia L, O'Farrell L (2005). Chemokine receptor CCR2 involvement in skeletal muscle regeneration. FASEB J.

[47] Ochoa O, Sun D, Reyes-Reyna SM, Waite LL, Michalek JE, McManus LM (2007). Delayed angiogenesis and VEGF production in CCR2−/− mice during impaired skeletal muscle regeneration. Am J Physiol Regul Integr Comp Physiol.

[48] Contreras-Shannon V, Ochoa O, Reyes-Reyna SM, Sun D, Michalek JE, Kuziel WA (2007). Fat accumulation with altered inflammation and regeneration in skeletal muscle of CCR2-/- mice following ischemic injury. Am J Physiol Cell Physiol.

[49] Godfrey C, Muses S, McClorey G, Wells KE, Coursindel T, Terry RL (2015). How much dystrophin is enough: the physiological consequences of different levels of dystrophin in the mdx mouse. Hum Mol Genet.

[50] Terashima Y, Onai N, Murai M, Enomoto M, Poonpiriya V, Hamada T (2005). Pivotal function for cytoplasmic protein FROUNT in CCR2-mediated monocyte chemotaxis. Nat Immunol.

[51] Devreotes P, Janetopoulos C (2003). Eukaryotic chemotaxis: distinctions between directional sensing and polarization. J Biol Chem.

[52] De Becker A, Riet IV (2016). Homing and migration of mesenchymal stromal cells: how to improve the efficacy of cell therapy?. World J Stem Cells.

[53] Liang F, Giordano C, Shang D, Li Q, Petrof BJ (2018). The dual CCR2/CCR5 chemokine receptor antagonist Cenicriviroc reduces macrophage infiltration and disease severity in Duchenne muscular dystrophy (Dmdmdx-4Cv) mice. PLoS One.

